# Electrical Design and Evaluation of Asynchronous Serial Bus Communication Network of 48 Sensor Platform LSIs with Single-Ended I/O for Integrated MEMS-LSI Sensors

**DOI:** 10.3390/s18010231

**Published:** 2018-01-15

**Authors:** Chenzhong Shao, Shuji Tanaka, Takahiro Nakayama, Yoshiyuki Hata, Masanori Muroyama

**Affiliations:** 1 Department of Robotics, Tohoku University, Miyagi 980-8579, Japan; tanaka@mems.mech.tohoku.ac.jp; 2 Microsystem Integration Center, Tohoku University, Miyagi 980-8579, Japan; muroyama@mems.mech.tohoku.ac.jp; 3 Partner Robot Div., Toyota Motor Corporation, Toyota, Aichi 470-0309, Japan; takahiro_nakayama_aa@mail.toyota.co.jp; 4 System & Electronics Engineering Dept. III, Toyota Central R&D Labs., Inc., Nagakute, Aichi 480-1192, Japan; yhata@mosk.tytlabs.co.jp

**Keywords:** robot tactile sense, dedicated CMOS-LSI, LSI network system, data collision avoidance, backbone bus topology, single-ended signaling

## Abstract

For installing many sensors in a limited space with a limited computing resource, the digitization of the sensor output at the site of sensation has advantages such as a small amount of wiring, low signal interference and high scalability. For this purpose, we have developed a dedicated Complementary Metal-Oxide-Semiconductor (CMOS) Large-Scale Integration (LSI) (referred to as “sensor platform LSI”) for bus-networked Micro-Electro-Mechanical-Systems (MEMS)-LSI integrated sensors. In this LSI, collision avoidance, adaptation and event-driven functions are simply implemented to relieve data collision and congestion in asynchronous serial bus communication. In this study, we developed a network system with 48 sensor platform LSIs based on Printed Circuit Board (PCB) in a backbone bus topology with the bus length being 2.4 m. We evaluated the serial communication performance when 48 LSIs operated simultaneously with the adaptation function. The number of data packets received from each LSI was almost identical, and the average sampling frequency of 384 capacitance channels (eight for each LSI) was 73.66 Hz.

## 1. Introduction

Sensor networks are a well-known concept that a huge amount of sensors are embedded in buildings, machines and other items, and sensing data are collected to form “big data”. In the future, a variety of applications using a lot of sensors in a system will appear. In such a system, each sensor is wire-connected to a host or main computer, which is often connected to internet wirelessly. In most of such applications, the sensors should be as compact as possible including wirings, and the sensor system should be as simple as possible. This is often the case with a robot because it needs a lot of sensors but has a limited room for the sensor system inside the body.

For example, there have been intensive efforts to install many tactile sensors in robots so far, because future robots with human-like tactile sensation will have more natural and seamless interaction with us, which enables safer, more reliable and more accurate human-robot communication. To install a large-scale array of tactile sensors or other sensors in a robot, two readout schemes have been mainly investigated.

One scheme is to use a scanning readout circuitry [1]. The digitization of sensor output is performed off the sensor array in a central processing unit. To date, some scanning-readout type sensor arrays, which can include many sensing elements based on capacitive and resistive transduction, were reported [2,3,4,5,6,7]. However, the number and length of connecting wires increase with the number of sensing elements and the covering area. The interference effects along a long signal propagation pathway [8], the crosstalk from element circuits [9,10] and the long scanning time make this scheme difficult to scale efficiently for large-scale robotic sensor networks [1].

Another scheme is to digitize the sensor output at the site of sensation, and build a sensor network system with serial bus communication. This solution has advantages in reducing the amount of wiring [11,12], the interference effects and the crosstalk. However, the long sensor data conversion time of an analog-to-digital converter chip limits the system performance [13]. For example, Refs. [11,12] reported data conversion times of 8.73 ms and 1.6 ms, respectively, for tactile sensors based on the conventional serial bus communication.

**Figure 1:**
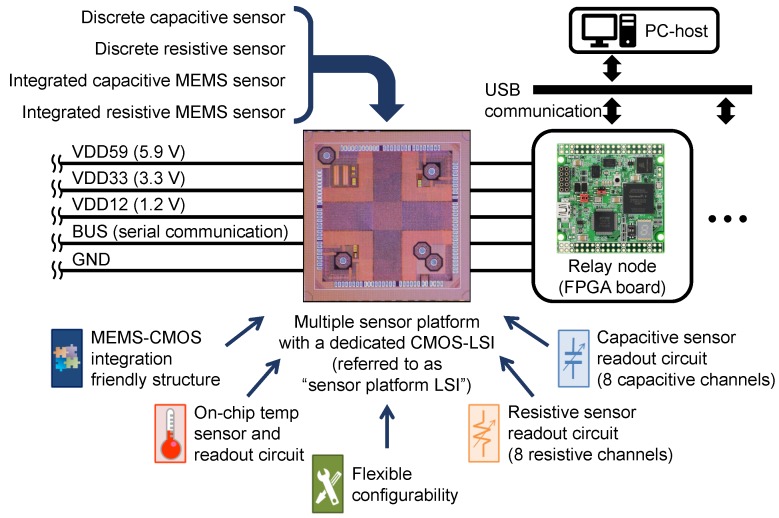
Serial-bus-communication-based sensor network system with multiple sensors using the sensor platform Large-Scale Integration (LSI).

In our previous research [14,15], we have designed a dedicated Complementary Metal-Oxide-Semiconductor (CMOS) Large-Scale Integration (LSI) (referred as “sensor platform LSI”) for bus-networked Micro-Electro-Mechanical-Systems (MEMS)-LSI integrated tactile, proximity or shock sensors, for which event-driven operation is convenient. Simple designs in bus communication scheme, protocol and sensor functions were adopted to make the die size smaller than 8 mm^2^ for a small pitch of sensor installation. The sensor platform LSI can be also connected with varieties of commercially-available capacitive (range from 1 pF to 10 pF) and resistive (range from 100 Ω to 1 MΩ) sensors [16] as shown in Figure 1. The detail of the sensor platform LSI is described in Section 2.2.

The serial-bus-communication-based sensing system using the sensor platform LSI is beneficial, especially when the number of sensors is large, for example, several tens or more. In such a situation, data collision and congestion in serial bus communication is a problem to address. To relieve this problem, the sensor platform LSI is equipped with collision avoidance, adaptation and event-driven functions. However, we have not connected more than 20 LSIs on a serial bus line in the previous study [15]. In this study, therefore, we designed and prototyped a serial bus network system with 48 sensor platform LSIs, and investigated the serial communication performance with the adaptation function.

This paper is organized as follows. Section 2 explains the structure of the network system of 48 sensor platform LSIs as well as the collision avoidance, adaptation and event-driven functions of the sensor platform LSI. Section 3 explains the design of the serial bus network on a Printed Circuit Board (PCB). Section 4 describes the evaluation of the designed serial bus network where 48 sensor platform LSIs were connected.

## 2. Sensor Platform LSI Network System

### 2.1. System Overview

**Figure 2:**
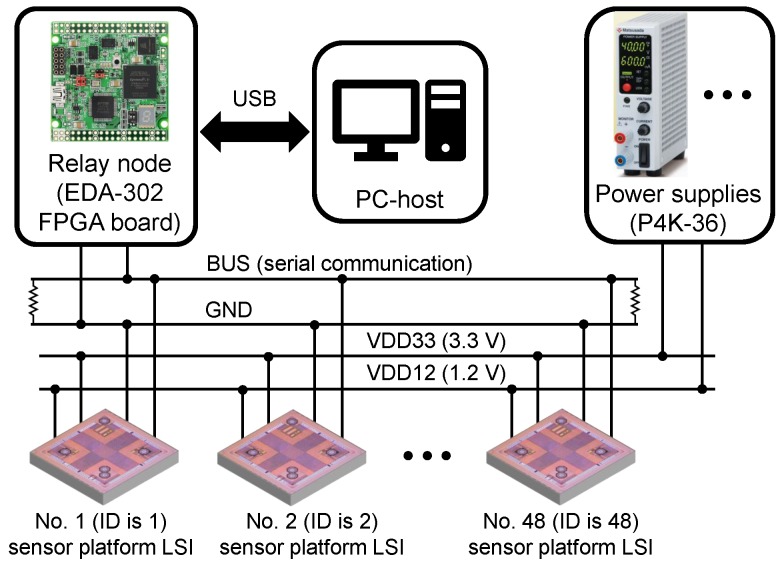
Network system with 48 sensor platform LSIs.

Figure 2 shows the overview of the network system, in which 48 sensor platform LSIs are implemented on a microstrip-based single-ended bus line. Power supplies provide 3.3 V (VDD33) and 1.2 V (VDD12) powers for each sensor platform LSI. The signal (BUS) and ground (GND) lines are connected to a relay node based on an Field Programmable Gate Array (FPGA).

The relay node performs some primary data processing, such as data decoding and error check using Cyclic Redundancy Check (CRC) codes, for data packets received from the sensor platform LSIs, and then transmits data packets to the PC-based host. The relay node also processes command packets received from the host, and then transmits the encoded command packets to a specific or all sensor platform LSIs. An 8-bit physical address is assigned to each sensor platform LSI for its identification and specific configuration. The address with all bits at ‘1’ is reserved for broadcast. The relay node communicates with the host through a USB interface (FT2232HL chip from Future Technology Devices International Ltd., Glasgow, UK).

The host was programmed using C# language on a Personal Computer (PC) to analyze data packets received from the relay node as well as to send configuration command packets to the sensor platform LSIs through the relay node. The communication between the host and the relay node is based on USB, which is supported by FT2232HL chip on the relay node and a Dynamic Link Library (DLL) file provided by Future Technology Devices International Ltd. on the PC.

### 2.2. Sensor Platform LSI

**Figure 3:**
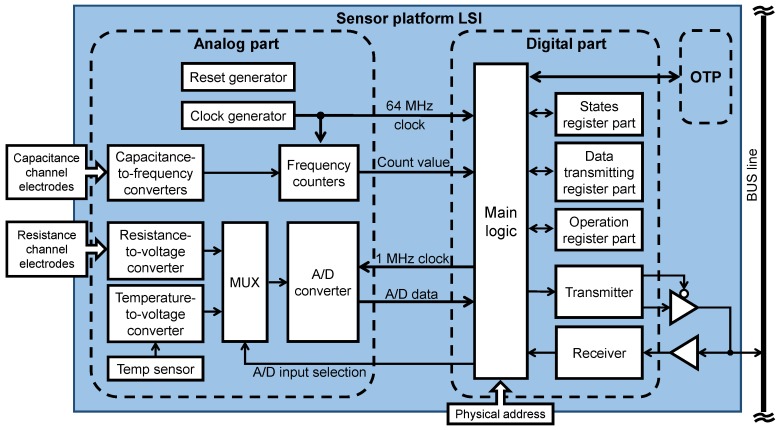
Block diagram of the sensor platform LSI.

The sensor platform LSI was fabricated by a TSMC (Taiwan Semiconductor Manufacturing Co., Ltd., Hsinchu, Taiwan) 0.13 μm standard CMOS process technology. As shown in Figure 3, the analog part includes a reset generator, a clock generator, capacitance-to-frequency converters, frequency counters, a resistance-to-voltage converter, an Analog-to-Digital Converter (ADC) and a diode-based temperature sensor. The on-chip clock is generated by a ring oscillator circuit, and its typical frequency is 64 MHz in design (about 61 MHz in measurement). The sensor platform LSI operates according to this internal clock.

The digital part includes main logic, data transmitting and command receiving circuits, and three function register blocks. The voltage levels of physical address pads can be adjusted to set the physical address of a specific sensor platform LSI. To relieve data collision and congestion, we implemented the collision avoidance, adaptation and event-driven functions [17].

The nonvolatile One-Time Programmable (OTP) memory stores the configuration information of the sensor platform LSI such as its own ID number, threshold values and sampling time. However, we did not use the OTP memory in this study. Thus, in the network system, there was no 5.9 V (VDD59) power line that was used for writing the OTP memory.

Each sensor platform LSI can be configured to operate in either capacitive or resistive sensing mode. In capacitive sensing mode, the sensor platform LSI processes signals from eight capacitance channels. The capacitance readout circuit is composed of a capacitance-to-frequency converter and a frequency counter. For precise sensing and fast processing, eight sets of the converter and counter were used for eight capacitance channels, which operate simultaneously. Each capacitance channel can connect a capacitive sensor with a capacitance ranging from 1 pF to 10 pF [15]. The typical frequency resolution of the capacitance-to-frequency converter is 56.6 kHz/fF at 1 pF, 3.8 kHz/fF at 5 pF, 1.0 kHz/fF at 10 pF. The capacitance of our MEMS tactile sensor [18] ranges from 0.9 pF to 1.6 pF, in which the frequency resolution is high. The bus communication speed can be configured to be 1 MHz, 2 MHz, 4 MHz, 8 MHz, 16 MHz or 32 MHz by dividing the on-chip clock (64 MHz in design) for different types of data receiving devices.

For signal transmitting method, there are single-ended and differential signaling. The latter, which is employed by standards like Low-Voltage Differential Signaling (LVDS), Current Mode Logic (CML), RS485 and so on, is more robust against electrical noise than the former. However, the current sensor platform LSI has adopted a single-ended signaling I/O cell for simplicity.

For asynchronous communication with the relay node, we used Non-Return-to-Zero Inverted (NRZI) encoding to map a binary signal to a physical signal and 4B5B to avoid a long series of zeros. We also used unique data and command packet formats with variable length. The asynchronous serial bus communication between the sensor platform LSI and the relay node was supported by the delay window blind oversampling clock and data recovery algorithm [19,20]. In the following subsections, the functions that are implemented in the sensor platform LSI and important in this study, i.e., collision avoidance, adaptation function, event-driven function and configuration, are explained.

#### 2.2.1. Collision Avoidance

To implement Carrier Sense Multiple Access in addition of Collision Avoidance (CSMA/CA), we used the dual-driving regular I/O cell (CMOS type) with Schmitt-trigger input, which enables the sensor platform LSI to send data packets on the bus and listen to the bus line simultaneously. The nominal values of the low to high and high to low threshold values of the Schmitt-trigger are 1.54 V and 1.17 V, respectively. Before sending a data packet on the bus, a sensor platform LSI checks the bus status for four cycles in its data sending clock (e.g., 4 μs in 1 MHz transmission speed). During these four checking cycles, if the bus logic level judged by the LSI’s I/O cell is logic 0, the LSI regards the bus status as free, or busy otherwise. When the bus status is considered as free by the LSI, it starts sending the data packet. When the bus status is considered as busy by the LSI, it will wait for a certain time interval according to the adaptation function explained in Section 2.2.2. In addition, the maximum number of continuous zeros in a data packet is 2 because of the 4B5B encoding. Therefore, a sensor platform LSI doesn’t have access to the shared bus, when the bus is transmitting a data packet.

**Figure 4:**
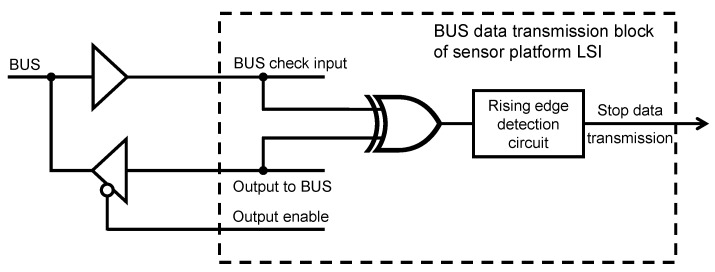
Schematic of collision detection circuit.

When many sensor platform LSIs operate simultaneously on a shared bus line with the same data transmission speed, several LSIs may start checking the bus status at the same time. Then, the bus status may be regarded as free by two or more LSIs that will start sending data packets at the same time, and data collision will happen. To reduce data collision, the sensor platform LSI performed the collision avoidance function to stop sending data packets once the data collision happens. Then, the bus line can soon be spared for transmitting other data packets. To perform the collision avoidance function, the LSI samples the bus voltage level at every positive edge of the on-chip clock (64 MHz in design) when sending data packets. At the sampling point, if the current bus logic level judged by the LSI’s I/O cell is not the same as the logic level which the LSI sent on the bus, the LSI regards this situation as data collision and stops sending data. As shown in Figure 4, when the “BUS check input” and “Output to BUS” are in different logic levels, the exclusive OR (XOR) gate generates a rising edge which stops data transmission by a rising edge detection circuit. We adopted one of the simplest data collision avoidance scheme mentioned above and will use this sensor platform for the applications where critical real time control is not required.

#### 2.2.2. Adaptation Function

**Figure 5:**
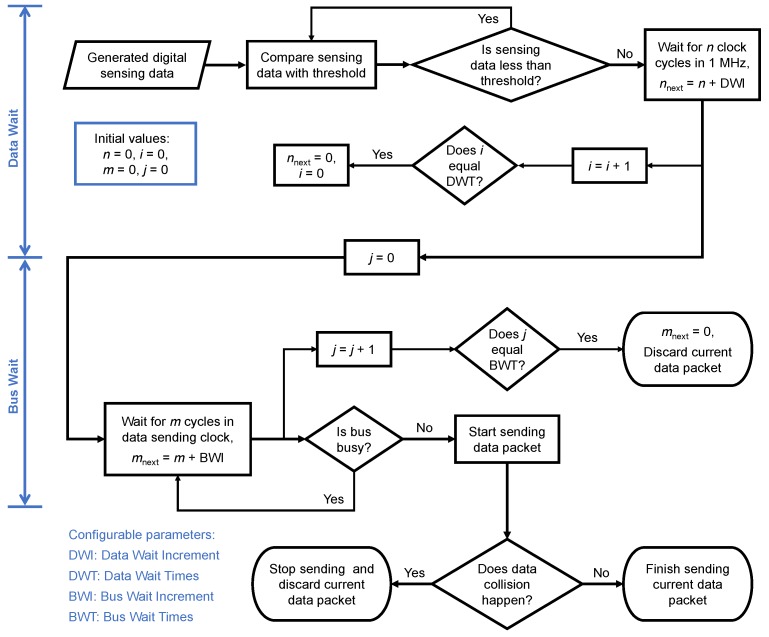
Adaptation function of sensor platform LSI.

For relieving data congestion, the sensor platform LSI has a simple adaptation function to reduce the number of data packets sent by a specific LSI and avoid a specific LSI from being dominant in the serial bus line. As shown in Figure 5, this function includes two processes: “Data Wait” and “Bus Wait”. In the “Data Wait” process, when the digital sensing data exceeds the threshold value, a data sending start signal waits for a certain number of clock cycles (*n*) in 1 MHz. With the increase in the number of data packets (*i*) that enter this process, *n* increases linearly with an increment of “Data Wait Increment” that is configurable. When *i* reaches the set value of the configurable “Data Wait Times”, *n* and *i* are reset to 0.

After *n* clock cycles in 1 MHz, the data sending start signal waits for another certain number of cycles (*m*) in a data sending clock, and the number of attempts (*j*) to send the data packet is reset to 0. After *m* clock cycles, the LSI attempts to send the data packet by checking the bus status for four cycles in its data sending clock (e.g., 4 μs in 1 MHz transmission speed). If the bus logic level judged by the LSI’s I/O cell is logic 0 during these four checking cycles, the LSI regards the bus status as free, or busy otherwise. When the bus is free, the sensor platform LSI starts sending the data packet. When the bus is busy, the sensor platform LSI will try to send the same data packet again after $m_{next}$ cycles in its data sending clock. With the increase in the number of attempts (*j*) to send the data packet, *m* increases linearly with an increment of “Bus Wait Increment” which is configurable. When *j* reaches the set value of the configurable “Bus Wait Times”, *m* is reset to 0, the data packet is discarded and a new data packet enters the “Data Wait” process. When a data collision happens during a packet transmission, the sensor platform LSI stops sending data immediately. The suspended data packet is discarded and a new data packet comes to the “Data Wait” process.

#### 2.2.3. Event-Driven Function

For further relieving data congestion, the sensor platform LSI has the event-driven function to reduce the number of sensor platform LSIs that attempt to send data packets on the bus line. According to our design, we can configure the threshold value for each capacitance or resistance channel of the sensor platform LSI. When a sensor connected to a channel of a sensor platform LSI is stimulated, the converted digital value from the channel changes. If the digital value exceeds the configured threshold value of the channel, the sensor platform LSI generates a data packet including sensing data from all the eight capacitance or resistance channels, and tries to send the data packet on the bus. We adopted this strategy because our MEMS tactile sensor uses six or eight channels individually. Smarter event-driven functions such as adaptive threshold [21] and send-on-delta system [22] were reported, but the simple one used in this study is still useful to reduce the number of packets on the serial bus.

**Figure 6:**
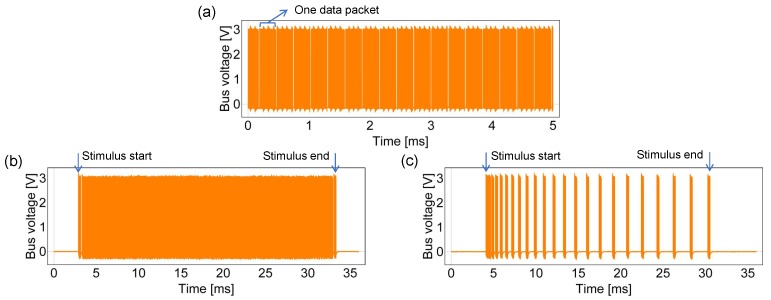
Bus status with different function configurations: (**a**) with neither adaptation nor event-driven function; (**b**) with event-driven function; (**c**) with adaptation and event-driven functions.

Figure 6 shows the signals of the bus on which one sensor platform LSI is operating with different configurations. When neither adaptation nor event-driven function is turned on (Figure 6a), the bus is busy with a small idle time ratio. By enabling event-driven (Figure 6b) and adaptation (Figure 6c) functions in order, the bus idle time ratio becomes larger. For the adaptation function configuration in Figure 6c, “Data Wait Increment”, “Data Wait Times”, “Bus Wait Increment” and “Bus Wait Times” were configured to 80, 64, 1 and 16, respectively. Note that Figure 6 shows the effects of the adaptation and event-driven functions when only one LSI operates to help illustrate these two functions. In the experiment, only the adaptation function was used.

#### 2.2.4. Configuration

**Figure 7:**
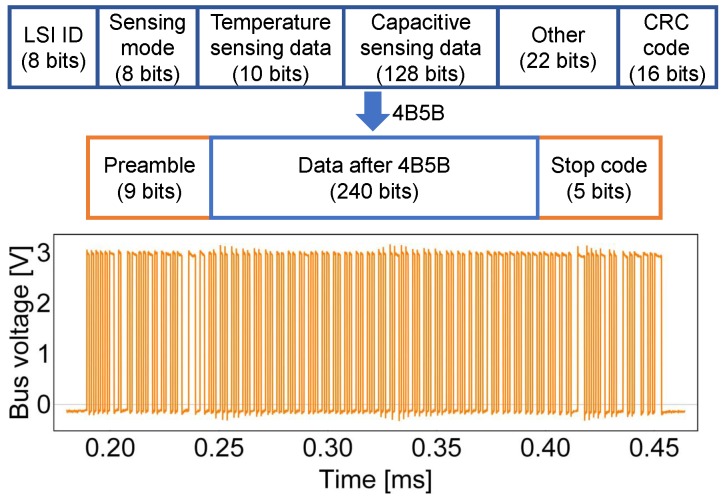
Structure and waveform of a data packet.

The physical address is used to send commands to a specific sensor platform LSI to write its registers for configuration. In this study, all sensor platform LSIs were configured to operate in capacitive sensing mode. As shown in Figure 7, one data packet was configured to include 24 bytes of data: ID number (8 bits), sensing mode (8 bits), sensing data from the on-chip temperature sensor (10 bits), sensing data from capacitive channels (128 bits), other LSI status information (22 bits) and CRC code (16 bits). After 4B5B encoding and attaching a 9-bit preamble and a 5-bit stop code, one data packet consists of 254 bits. Assuming the frequency of the on-chip clock is 61 MHz and the data transmission speed is 61/64 MHz = 953.1 kHz, the length of one data packet is 266.5 μs.

## 3. Design of Serial Bus Network System

### 3.1. Design of Experimental Setup

We designed a serial bus network system on a PCB composed of unit network systems with 16 sensor platform LSIs. In this study, we used the PCB to construct the experimental setup, because the design and production of PCBs are well established and easy to access, which can provide a stable evaluation environment. Due to this reason, the PCB is convenient to investigate the behavior of the sensor platform LSIs on the serial bus line as the first trial. According to Ref. [23], the design method of this PCB-based network system can be partially applied to the development of the network system based on flexible circuits.

**Figure 8:**
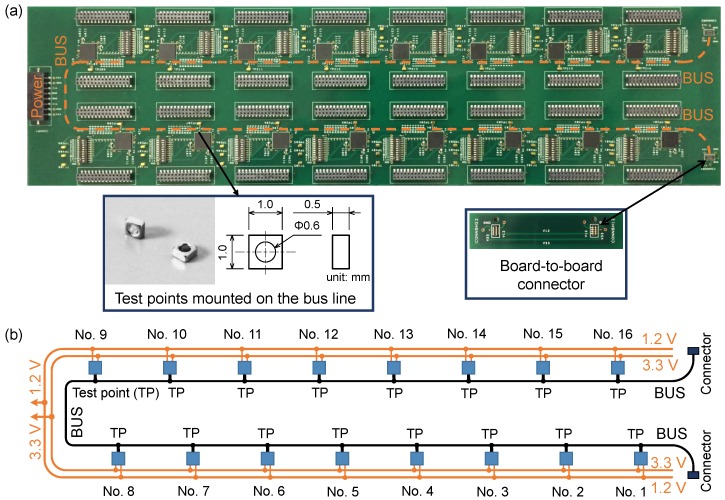
Unit network system with 16 sensor platform LSIs for building larger network system: (**a**) photograph; (**b**) schematic diagram.

Figure 8 shows the photograph and schematic of the unit network system made on a PCB. On this board, 16 sensor platform LSIs were mounted on an 80 cm long bus line in backbone topology with short stubs. The package of the sensor platform LSI is Quad Flat No-leads (QFN) package with 64 terminals. There were two power lines (1.2 V and 3.3 V) on the PCB. Fifteen capacitors of 1 μF were added to the 3.3 V power line for power stabilization. Eight switches were used to adjust the physical address of a sensor platform LSI. To measure the signal waveform sent by each sensor platform LSI near the corresponding bus pin, a small Test Point (TP) was mounted on the junction point at which the stub connecting the sensor platform LSI meets the backbone bus line.

**Figure 9:**
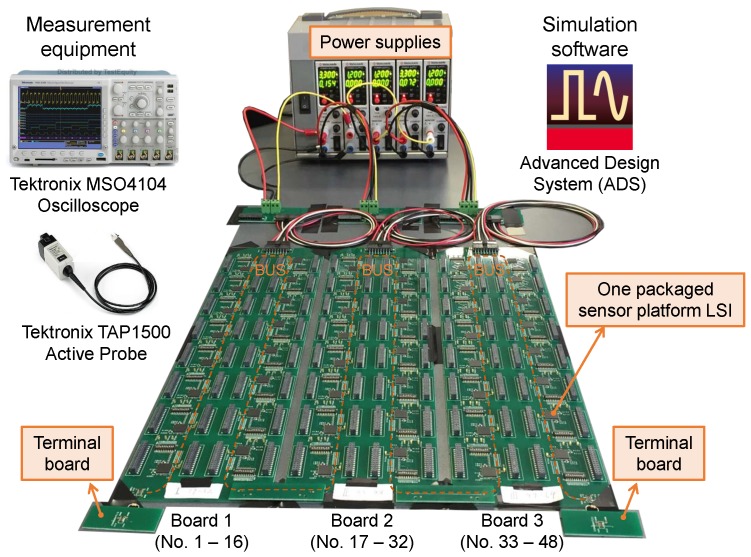
Photograph of network system with 48 sensor platform LSIs.

The serial bus network system with 48 sensor platform LSIs was constructed using three unit network systems shown in Figure 9. To connect the PCBs, we designed a board-to-board connector, through which the signal and ground lines of two PCBs were connected. The bus line cross section of the connector is the same as the unit network system to reduce the discontinuity of the bus line characteristic impedance. For the bus line termination, we designed a terminal board, on which a terminal resistor was soldered. Five DC power supplies were used to supply 1.2 V and 3.3 V powers for three PCBs. A bus communication speed of 1 MHz was selected in this study because the maximum available communication speed was unknown for a long serial bus line with a lot of sensor platform LSIs connected.

For the bus line design, we used Advanced Design System (ADS) simulation software by Keysight Technologies, Inc. (Santa Rosa, CA, USA) to analyze the signal integrity of the sensor platform LSI. The Input/output Buffer Information Specification (IBIS) provided by TSMC was used to simulate the operation of the LSI. To measure bus signal waveforms, we used an active probe with an input capacitance less than 1 pF (TAP1500, Tektronix, Inc., Beaverton, OR, USA).

### 3.2. Design of Characteristic Impedance and Termination of Serial Bus

After connecting three unit network systems together, the length of the bus line becomes 2.4 m. A long bus line means a long returning time of reflected signals from the terminals back to the LSI’s I/O cell that drives the bus. Since the LSI has the collision avoidance function, the LSIs at some positions on a long bus line (around the middle part) with low characteristic impedance may never send normal data packets.

**Figure 10:**
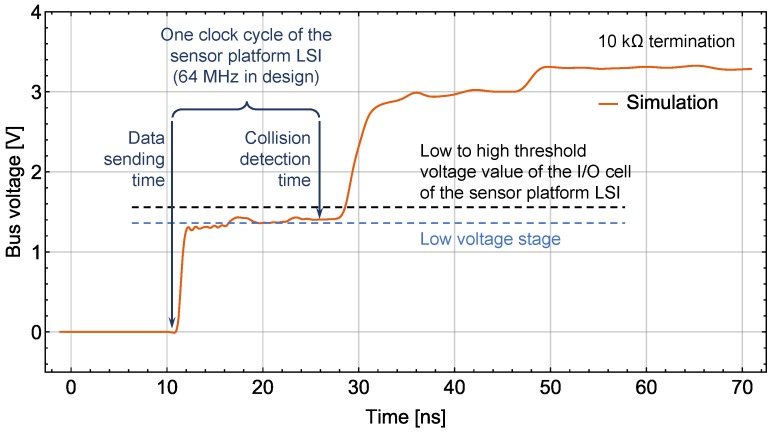
Simulated waveform of a logic 1 rising edge.

Figure 10 shows a simulated waveform of the rising edge of a logic 1 signal recorded at the TP nearest to the bus pin of the middle sensor platform LSI (No. 24 LSI) that sends this signal. A lower bus line characteristic impedance results in a lower value of the “Low voltage stage”. A longer bus line results in a longer lasting time of the “Low voltage stage”. In the case shown in Figure 10, the bus line characteristic impedance is adjusted to a low value (35.5 Ω at 1 MHz calculated by ADS). This low characteristic impedance makes the bus logic level judged by the LSI’s I/O cell different from the logic level that the LSI sent on the bus. Therefore, the LSI will regard this situation as data collision and stop sending the data packet. This can happen to each logic signal sent by this LSI, and it will never send a normal data packet. To avoid this, the bus line characteristic impedance needs to be increased to lift the “Low voltage stage” over the low to high threshold voltage value of the I/O cell of the LSI. As for the falling edge, the high characteristic impedance of the bus line works in a similar way.

When the LSI drives the bus, its I/O cell’s equivalent inner resistance is around 20 Ω, which causes a certain fraction of the voltage drop. In addition, the typical capacitance of the bus pin of the LSI is 2.83 pF. This bus pin capacitance decreases the equivalent characteristic impedance of the bus line. Therefore, the characteristic impedance of the microstrip bus line was tuned to 127.6 Ω at 1 MHz and 112.2 Ω at 1 GHz based on Finite Element Method (FEM) simulation (FEMTET from Murata Software Co., Ltd., Tokyo, Japan).

A nearly matched terminal resistance (110 Ω) is better in terms of signal reflection at the ends of the serial bus. However, a low terminal resistance leads to a large bus driving current, which results in a large vibration of the bus driving current when an LSI sends a data packet. This makes the 3.3 V power at the site of the LSI that sends data packets unstable, when many LSIs (e.g., more than 16) are connected to the same long (around 1 m) 3.3 V power line. The unstable 3.3 V power can lead to an abnormal behavior of the LSI. On the other hand, a large terminal resistance (10 kΩ) leads to a low bus driving current, which results in a low vibration of the bus driving current when an LSI sends a data packet. This helps to stabilize the 3.3 V power at the site of the LSI that sends data packets.

Therefore, we considered two cases of termination: 110 Ω (nearly matched) and 10 kΩ terminations. Simulation was done for each case. If the number of connected LSIs is relatively small, or the number of LSIs triggered at the same time is small (i.e., event-driven mode), 110 Ω may be a better choice for high speed communication because there is no severe bus signal fluctuation. In this study, however, we investigated one of the worst cases, i.e., all LSIs were triggered at the same time. Thus, we used 10 kΩ resistors as terminals. The terminal mismatch leads to the vibration of the bus signal. However, because of the high bus characteristic impedance, the bus signal vibrates over or under the threshold value of the LSI’s I/O cell. Thus, we think the high terminal resistance does not have negative impact on the communication performance under 1 MHz data transmission speed.

## 4. Evaluation of Serial Bus Network System

### 4.1. Signal Waveform

**Figure 11:**
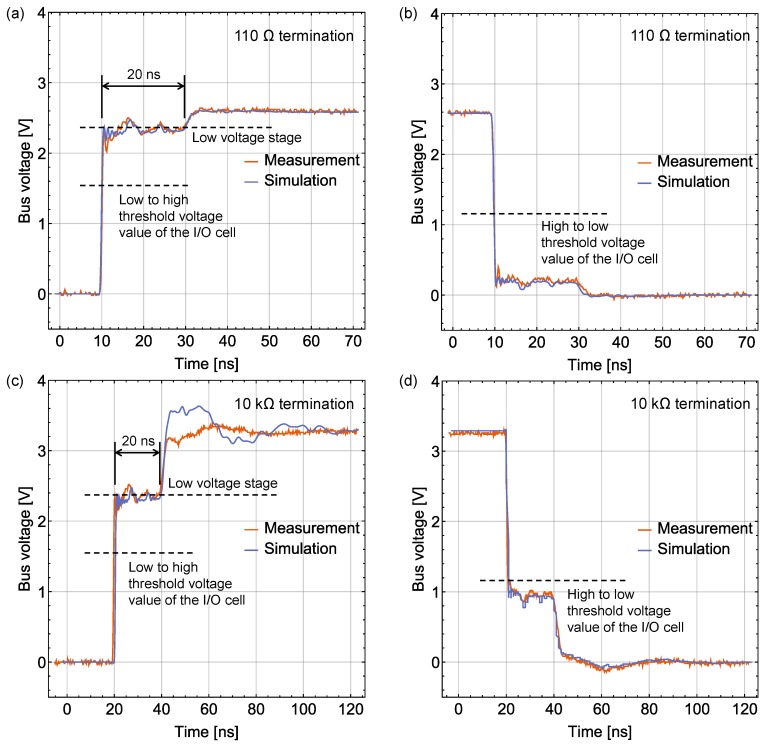
Signal waveform sent by the middle sensor platform LSI and recorded near its bus pin: (**a**) rising edge and (**b**) falling edge under 110 Ω termination; (**c**) rising edge and (**d**) falling edge under 10 kΩ termination.

Bus signal waveform was measured at the TP nearest to the bus pin of the LSI that sends data packets. Figure 11 shows the measured and simulated waveforms when the middle sensor platform LSI (No. 24 LSI) operated. Data sending by this LSI is one of the severest cases because of the longest returning time of the reflected signal from a terminal back to the bus pin. The high bus characteristic impedance makes the signal rises and falls through the corresponding threshold values shortly, which do not trigger the collision avoidance function. If we use a low bus characteristic impedance, such as the case shown in Figure 10, the around 20 ns “Low voltage stage” can cause constant triggering of the collision avoidance function when the LSI sends data packets.

As shown in Figure 11a,b, there is no severe bus signal fluctuation under nearly matched termination (110 Ω), which makes it a better choice for high speed communication when a small number of LSIs operate simultaneously. When the bus line is terminated by 10 kΩ resistors (Figure 11c,d), the bus signal vibrates over or under the threshold value of the LSI’s I/O cell, which does not have a negative impact on the communication performance under 1 MHz data transmission speed.

### 4.2. Serial Communication Analysis with Adaptation Function

For each sensor platform LSI, eight switches were used to set a specific physical address for configuration. Except for ID number, the configurations of all sensor platform LSIs were identical. The communication speed was set to 1 MHz. “Data Wait Increment”, “Data Wait Times”, “Bus Wait Increment” and “Bus Wait Times” were configured to 80, 16, 15 and 8, respectively. Threshold values of the event-driven function were set to zero.

**Figure 12:**
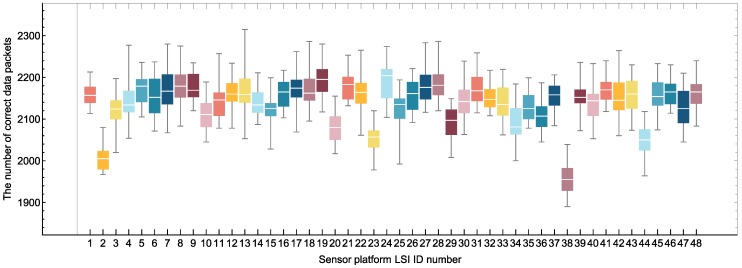
Number of correct data packets received in 30 s from each sensor platform LSI.

After sending configuration commands, 48 sensor platform LSIs operated simultaneously with the adaptation function. This is one of the worst cases in terms of data congestion on the bus because all sensor platform LSIs were triggered and tried to send packets simultaneously. We received data packets by the PC-host for 30 s every 5 min, and the data recording was continued for 110 min in total. CRC-16 was used to check the correctness of a received data packet. To illustrate the number of received correct data packets in 23 times of 110 min measurement sequences, a box whisker chart was drawn as shown in Figure 12. The whisker lower end, box bottom, band inside the box, box top and whisker upper end represent the minimum, first quartile, second quartile (median), third quartile and the maximum values of the numbers of correct data packets received in 23 timing slots, respectively. For all sensor platform LSIs, the number of received data packets per 30 s is between 1890 and 2315. For each sensor platform LSI, difference in the number of received data packets among different experiments is less than 12.76%.

**Figure 13:**
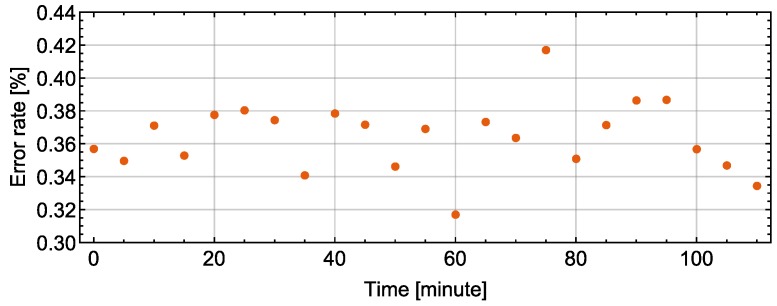
Error rate of the data packets received in 30 s from 48 sensor platform LSIs along with time.

These results confirm that Carrier Sense Multiple Access in addition of Collision Avoidance (CSMA/CA) was working as designed. As shown in Figure 13, the error rates of received data packets are less than 0.42% in 23 timing slots, which shows the reliability of our network system. Note that the residual error rate of CRC-16 designed in this study is on the order of $10^{-9}$, and negligible compared to the error rate of received data packets [24].

**Figure 14:**
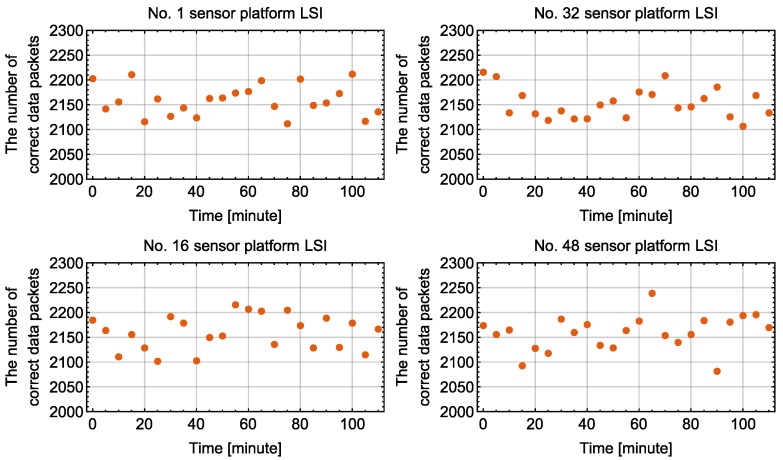
Number of correct data packets received in 30 s from a specific sensor platform LSI along with time.

Figure 14 shows the number of received data packets in 30 s along with elapsed time for four sensor platform LSIs. There is no obvious changing trend of the number of received data packets along with elapsed time, suggesting that the network system was stable.

**Figure 15:**
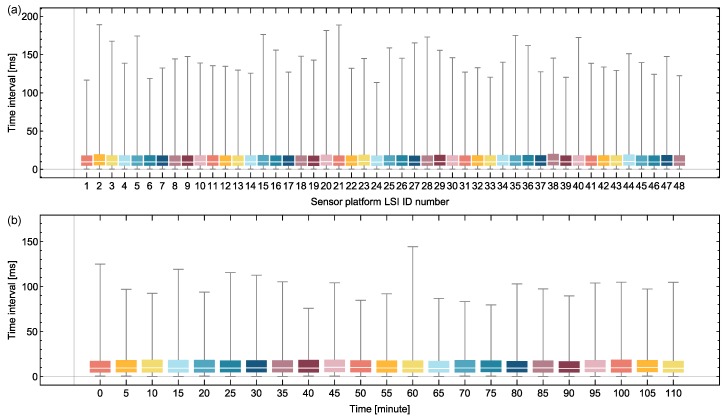
Time interval between two data packets received by the relay node successively from the same sensor platform LSI: (**a**) for 48 sensor platform LSIs within 30 s; (**b**) for a specific sensor platform LSI along with time.

To evaluate the sampling frequency of the network system, we extracted the time interval between two data packets received by the FPGA-based relay node successively from the same sensor platform LSI within 30 s. The time information was provided by the relay node, since the relay node attached a time stamp (in 0.1 μs) to each received data packet. Figure 15a shows the time intervals of 48 sensor platform LSIs in 30 s. Figure 15b illustrates the time intervals of No. 8 sensor platform LSI along with elapsed time in 23 timing slots. Among the time intervals of each sensor platform LSI extracted in each data receiving, the median is around 10 ms, and more than 75% of the time intervals are less than 21 ms.

Among the time intervals of all sensor platform LSIs extracted in 23 timing slots (2,361,558 values in total), the first quartile, median, third quartile and mean values are 4.85 ms, 9.76 ms, 18.39 ms and 13.58 ms, respectively. The minimum value is 267.2 μs, which means that there is a possibility that a sensor platform LSI sends two data packets successively. The maximum value is 189.1 ms which means that the worst sampling frequency is 5.3 Hz. Using the mean value, the average sampling frequency for all 384 capacitance channels (eight capacitance channels per sensor platform LSI) is 73.66 Hz.

**Figure 16:**
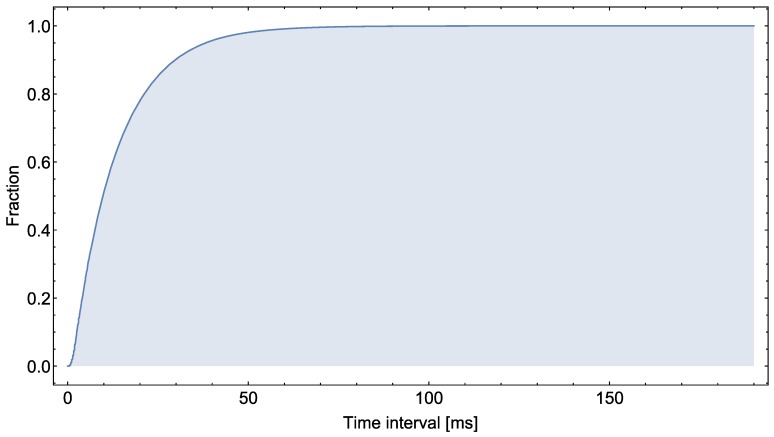
Empirical distribution function of time interval values.

The empirical distribution function of these measured time interval values is illustrated in Figure 16. This function is a step function that jumps up by 1/n at each of the *n* value points (n=2,361,558. Its value at a specified time interval value is the fraction of time interval values that are less than or equal to the specified value. As shown in Figure 16, 90.2% of the values are less than 30 ms. The fraction of values over 50 ms is less than 2%, and the fraction of values over 100 ms is less than 0.04%.

### 4.3. Discussion

We compared our network system of 48 sensor platform LSIs with other sensor systems mainly used for tactile sensation. There are eight capacitance channels and capacitance-to-digital converters in each sensor platform LSI. In total, 384 capacitance channels and capacitance-to-digital converters are included in this network system. The average sampling frequency of these capacitance channels is 73.66 Hz, which is higher than most of the systems shown in Table 1. The length of the bus line is 2.4 m, which shows an ability to cover much larger areas. Since each sensor platform LSI can connect eight capacitive sensors, the network system can include 384 capacitive sensors.

Assume that our network system has a scanning readout mode. We send a Send One Data Packet command to a specific sensor platform LSI, and the corresponding LSI receives this command and sends one data packet. Then, we receive this data packet, and send another Send One Data Packet command to the next LSI. According to this procedure, we scan all the 48 LSIs continuously. Assume that the Send One Data Packet command includes 32 bits of which eight bits are for the physical address, eight bits are for command identification and 16 bits are for CRC code. After 4B5B encoding, attaching a 9-bit preamble and a 5-bit stop code, the length of the command becomes 54 bits. Assume that there is a time interval between finishing receiving and starting to send, being four cycles of data or command transmission speed, and the data and command transmission speeds are the same as 61/64 MHz = 953.1 kHz. Then, sampling one LSI needs (254+54+4+4)/0.9531 μs = 331.55 μs. The constant sampling frequency of all 384 capacitance channels is 62.84 Hz. This frequency is 10 Hz less than the average sampling frequency of our real network system because of the command overhead. On the other hand, the stimuli of all the 48 LSIs is a rare case in real application. We think the flexibility of our network system, enabled by the adaptation and event-driven functions as well as many configurable parameters, makes it a useful network system in robot applications, which we will confirm as a next step.

## 5. Conclusions

In this study, we designed and constructed a serial bus network system with 48 sensor platform LSIs, which can be the platform of a sensor network covering a wide area with the minimum wiring. The CSMA/CA is enabled by the collision avoidance function of the LSI. To avoid false triggering of the collision avoidance function, the characteristic impedance of the bus line should be designed to be a relatively large value compared with the equivalent inner resistance of the LSI bus driver, especially in a long bus line application. After the design and fabrication of the network system on a PCB, we evaluated the serial communication performance of the network system when 48 sensor platform LSIs operated simultaneously with the adaptation function. We confirmed that the system worked as designed, with low error rate of received data packets. The number of received data packets from each sensor platform LSI was almost identical and stable throughout the evaluation. We also obtained an average sampling frequency as high as 73.66 Hz for each of 384 capacitance channels sampled via the same bus line. In conclusion, the network system of 48 sensor platform LSIs with a backbone bus topology stably operated as designed, and the adaptation function featuring this system worked effectively. This system is useful for sensor networks including but not limited to a tactile sensor network for robots.

## Figures and Tables

**Table 1:** Summary of some tactile sensor systems with their performance.

System Structure	Sensor Output Digitization Place	Force Sensing Principle	The Number of Capacitance or Resistance Channels	Covering Area	The Number of Analog-to-Digital Converters (ADC)	Sampling Frequency (Hz)	Reference
Tactile sensor array with scanning readout circuitry	Off the sensor array	Capacitive	8×8	1.6×1.6 ${\mathrm{cm}}^{2}$	1 (AD7153)	3	[2]
			8×8	3.6×3.6 ${\mathrm{cm}}^{2}$	1 (ADuC841)	n.a.	[3]
			16×16	2.2×2.2 ${\mathrm{cm}}^{2}$	1	20	[4]
		Resistive	8×8	4×4 ${\mathrm{cm}}^{2}$	1 (PIC18F2523)	16	[5]
			8×12	9×13 ${\mathrm{cm}}^{2}$	1 (dsPIC30F5015)	n.a.	[6]
			8×8	2.5×2.5 ${\mathrm{cm}}^{2}$	1 (dsPIC33FJ256)	100	[7]
Serial bus-based tactile sensor network	At the place of sensation	Capacitive	12×16	3.9×16 ${\mathrm{cm}}^{2}$	16 (AD7147)	25	[11]
			114	n.a.	114 (MPL115A2)	50	[12]
		Capacitive or resistive plus temperature sensing	8×48	2.4 m (bus line length)	8×48	73.66 (mean) 5.3 (worst)	This work
